# Inter-assay variability in anti–JC virus serology in the era of natalizumab biosimilars: implications for PML risk stratification

**DOI:** 10.3389/fimmu.2026.1818341

**Published:** 2026-07-29

**Authors:** Ana Belén Caminero, Celia Oreja-Guevara

**Affiliations:** 1 Department of Neurology, Complejo Asistencial de Ávila, Ávila, Spain; 2 Department of Neurology, Hospital Clínico San Carlos, IdISSC, Madrid, Spain; 3 Department of Medicine, Faculty of Medicine, Complutense University of Madrid (UCM), Madrid, Spain

**Keywords:** anti-JCV antibody index, biosimilars, inter-assay variability, JC virus, multiple sclerosis, natalizumab, progressive multifocal leukoencephalopathy, risk stratification

## Abstract

Anti–John Cunningham virus (JCV) serological testing constitutes a cornerstone of progressive multifocal leukoencephalopathy (PML) risk assessment in patients receiving natalizumab for multiple sclerosis (MS). Existing PML risk stratification algorithms were originally developed and validated within a single-assay framework based on the STRATIFY JCV™ DxSelect™ platform. The recent introduction of natalizumab biosimilars in Europe has coincided with implementation of IMMUNOWELL™ JCV IgG assay, creating a multi-assay environment in which quantitative antibody index values and binary serostatus classifications may not be directly interchangeable. We conducted a structured narrative review of comparative studies evaluating STRATIFY and IMMUNOWELL performance in natalizumab-treated cohorts. Outcomes included seroprevalence, qualitative concordance, quantitative index distribution, reclassification across established PML risk categories, and reported changes in clinical management. Across analytical validation datasets and real-world populations, IMMUNOWELL classified a higher proportion of individuals as JCV-positive than STRATIFY. Reported binary discordance ranged from approximately 6% to 38%, with a predominance of IMMUNOWELL-positive/STRATIFY-negative classifications. Longitudinal studies further showed that many low-positive IMMUNOWELL results fluctuated or reverted over time. The available evidence suggests that the transition from a mono-assay to a multi-assay testing environment introduces a previously underrecognized source of variability in PML risk stratification. In the absence of a biological gold standard for latent JCV infection, interpretation of discordant results should integrate antibody magnitude, longitudinal persistence, and clinical context rather than relying on binary thresholds.

## 1 Introduction

Natalizumab remains one of the most effective disease-modifying therapies (DMT) for relapsing–remitting multiple sclerosis (RRMS). Its long-term use, however, is constrained by the risk of progressive multifocal leukoencephalopathy (PML), an opportunistic central nervous system infection caused by reactivation of the John Cunningham virus (JCV) (1). Over the past decade, risk mitigation strategies have relied on anti-JCV antibody testing, incorporating both binary serostatus and quantitative antibody index values into widely adopted clinical algorithms (1, 2). These risk models were derived and calibrated using data generated exclusively with the STRATIFY JCV™ DxSelect™ assay (STRATIFY hereafter).

In 2023, the European approval of the first natalizumab biosimilar (Tyruko^®^) (3) introduced a new practical variable into this established framework. Alongside biosimilar implementation, an alternative commercial anti-JCV antibody platform, IMMUNOWELL™ JCV IgG (IMMUNOWELL hereafter), began to be used in routine practice. Clinical decision-making that was originally developed within a single-assay ecosystem is now occurring in a multi-assay context.

Although both STRATIFY and IMMUNOWELL are enzyme-linked immunosorbent assays (ELISA), they differ in calibration methods, analytical thresholds, and confirmatory procedures (4–9). These differences raise important questions regarding the interchangeability of quantitative index values and binary classifications across platforms. Assay-specific PML risk models for IMMUNOWELL have not yet been developed or prospectively validated.

This evolving analytical landscape introduces a structural challenge: clinical risk algorithms originally designed within a mono-assay paradigm are now being applied across assays with potentially distinct quantitative behavior near decision thresholds. As a result, discordance between assays may reflect analytical variability, biological variability, or a combination of both. Moreover, because no biological gold standard exists for latent JCV infection, the clinical significance of discordant results remains inherently difficult to determine. The implications of this shift extend beyond laboratory concordance and may influence categorical PML risk assignment and subsequent clinical management.

The present structured review aims to synthesize and critically examine the available comparative evidence between STRATIFY and IMMUNOWELL in natalizumab-treated populations. Particular emphasis is placed on overall seroprevalence differences, patterns of qualitative discordance, quantitative index distribution, reclassification across established PML risk categories, and the potential clinical management consequences resulting from inter-assay variability.

## 2 Methods

A structured narrative review of the literature comparing the STRATIFY JCV™ DxSelect™ (STRATIFY) and IMMUNOWELL™ JCV IgG (IMMUNOWELL) assays in patients with multiple sclerosis (MS) treated with natalizumab was conducted. The objective was to integrate, contextualize, and critically examine the available comparative evidence regarding assay performance and its potential implications for progressive multifocal leukoencephalopathy (PML) risk stratification and clinical decision-making within the current multi-assay environment.

**Figure 1:**
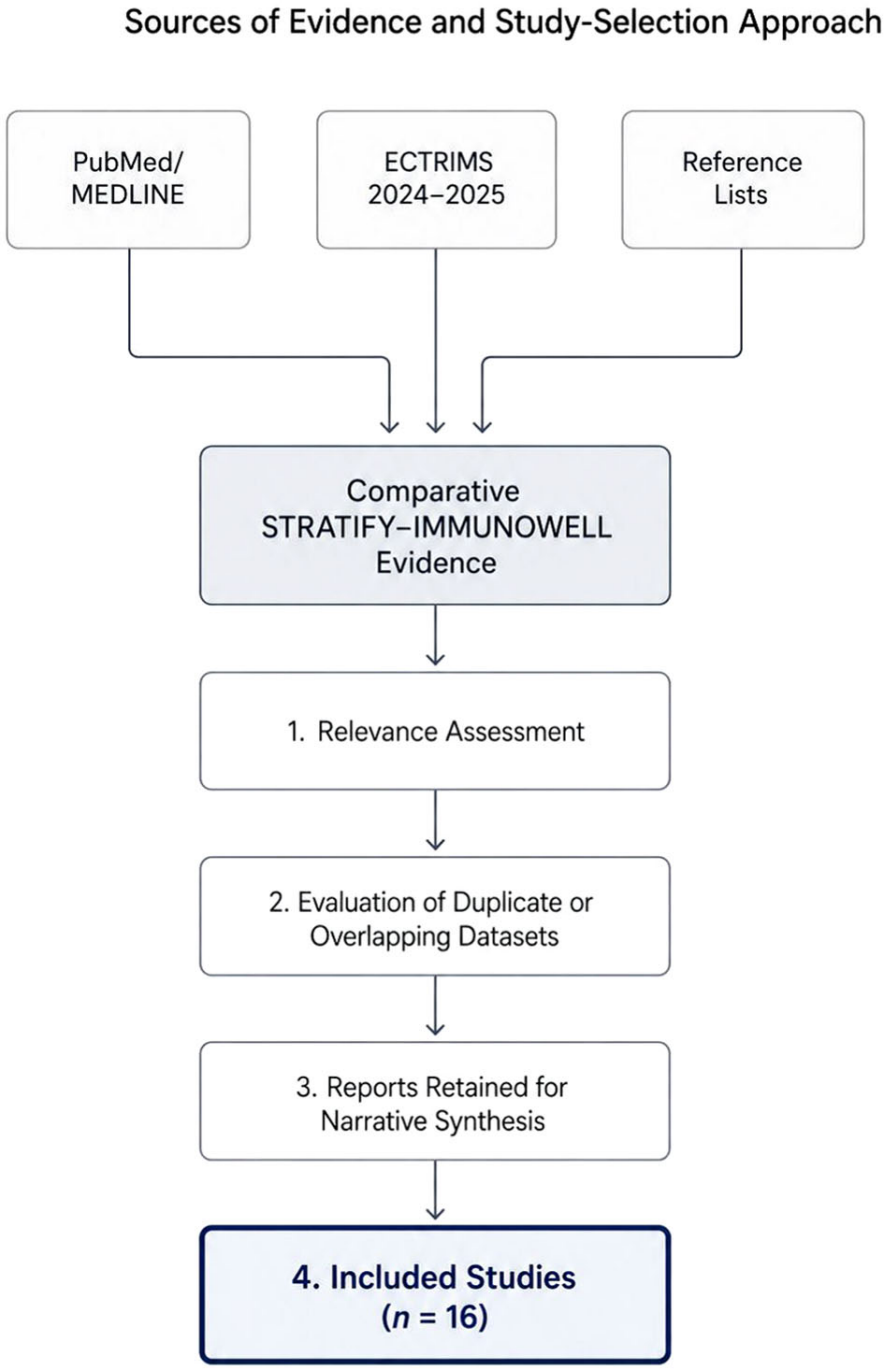
Sources of evidence and study-selection approach. Schematic representation of the structured narrative review process. Comparative evidence was identified through PubMed/MEDLINE searches, screening of ECTRIMS 2024–2025 congress abstracts, and review of reference lists from relevant publications. Retrieved reports underwent relevance assessment and evaluation for duplicate or overlapping datasets. When overlapping populations were subsequently reported as full peer-reviewed publications, journal articles were prioritised whenever possible. Studies providing direct comparative data between STRATIFY™ JCV DxSelect™ and IMMUNOWELL™ JCV IgG assays were included in the final narrative synthesis (n = 16).

The review followed a predefined structured search and study-selection approach aimed at identifying all available comparative evidence on STRATIFY and IMMUNOWELL assays in natalizumab-treated populations. Relevant literature was identified through focused searches of PubMed/MEDLINE using combinations of the following search terms: “natalizumab”, “JCV”, “JC virus”, “IMMUNOWELL”, “STRATIFY”, “biosimilar”, and “PML risk”. In addition, abstracts presented at recent European Committee for Treatment and Research in Multiple Sclerosis (ECTRIMS) congresses (2024–2025) were screened and reference lists from retrieved publications were reviewed to identify additional relevant reports. The literature search was designed to identify all available comparative reports published up to February 2026. No language restrictions were applied. **Figure 1** summarizes the sources of evidence and study-selection approach used in this review. This approach was designed to enhance transparency while remaining consistent with the narrative nature of the review.

Eligible sources included analytical validation studies, real-world cohort studies, observational studies, letters, and expert or consensus-oriented publications reporting direct comparisons between STRATIFY and IMMUNOWELL assays in natalizumab-treated populations. Studies were considered eligible if they reported paired, parallel, or sequential testing with both assays and provided comparative information on at least one of the following outcomes: (i) JCV seropositivity rates, (ii) qualitative agreement or discordance between assays, (iii) quantitative antibody index values, or (iv) reclassification across established index-based PML risk categories. Reports not providing direct comparative data beween assays were excluded.

Potential duplicate or overlapping datasets were carefully assessed. When conference abstracts and subsequent full publications reported identical populations and outcomes, only the peer-reviewed publication was retained for analysis. An exception was made when conference abstracts provided expanded cohorts, longer follow-up, or additional analyses not available in the corresponding publication. Conference abstracts were interpreted with caution given the limited methodological information typically available and the absence of full peer review.

Because included studies used different testing designs, including paired same-sample comparisons, parallel testing and sequential post-switch assessments, findings were interpreted taking into account the potential contribution of both analytical variability and biological fluctuations in anti-JCV antibody levels over time.

For each included source, study design, sample size, assay comparison methodology, and main results were extracted. When available, diagnostic performance measures, quantitative antibody index distributions, longitudinal observations, and reported changes in clinical management were also recorded. The level of evidence (peer-reviewed publication, conference abstract, or preprint) was also specified.

Given the heterogeneity in study design, populations, testing strategies, and outcomes reporting, findings were synthesized descriptively without statistical pooling. No formal risk-of-bias assessment was performed because this review was conceived as a structured narrative synthesis rather than a systematic review or meta-analysis.

All analyses were based exclusively on previously published or publicly presented data. No new patient data were collected, and no additional ethical approval was required.

## 3 Results

### 3.1 Overview of included studies

A total of 16 comparative reports were included in the narrative synthesis, comprising peer-reviewed publications, conference abstracts, and one preprint describing direct comparisons between STRATIFY and IMMUNOWELL assays in patients with MS treated with natalizumab (**Table 1**; **Figure 1**).

**Table 1:** Comparative studies evaluating STRATIFY and IMMUNOWELL assays in patients with multiple sclerosis treated with natalizumab.

Study (reference)	Country	Study category	Study design	Sample size (n)	Testing design	Key comparative findings	Level of evidence
Guerrieri et al. (4)	Italy	Real-world cohort	Cross-sectional	113	Parallel (same-day)	23.9% binary discordance; predominantly upward reclassification	Peer-reviewed
Vukusic et al. (5)	France	Real-world cohort	Multicentre retrospective	259	Parallel (same-day)	42.9% risk-category discordance; 3.9% high-risk upgrade; 10.8% potential treatment discontinuation	Peer-reviewed
Gelissen et al. (6)	Netherlands	Real-world cohort	Cross-sectional	94	Parallel (same-day)	Sensitivity 100%; specificity 57.5%; increased low-positive classifications with IMMUNOWELL	Peer-reviewed
Varley et al. (7)	United Kingdom	Post-switch cohort	Observational	497	Sequential	Sensitivity 98.8%; discordance concentrated in low-index range	Peer-reviewed
Varley et al. (8)	United Kingdom	Post-switch cohort	Expanded observational dataset	601	Sequential	58.7% of STRATIFY-negative patients reclassified as IMMUNOWELL-positive; sensitivity 99.0%; specificity 41.2%	Conference abstract
Høgestøl et al. (9)	Norway	Post-switch cohort	Real-world observational	116	Sequential	Increased seropositivity after switch; predominance of low-index positivity	Preprint
Adler et al. (10)	Germany	Analytical validation	Assay validation study	328 (cut-off cohort); 888 (clinical performance cohort)	Parallel	Sensitivity 97.2%; specificity 74.1%	Conference abstract
Azevedo et al. (11)	Germany	Analytical validation	Two-laboratory concordance study	493 samples / 496 patients	Parallel	Sensitivity 93%; specificity 73%; JCV seroprevalence 50.3%	Conference abstract
Chisari et al. (12)	Italy	Real-world cohort	Single-centre cross-sectional	120	Parallel	κ = 0.86; highest agreement at higher antibody index values	Peer-reviewed
Garelli et al. (13)	Italy	Post-switch cohort	Sequential reassessment study	100 (75 retested)	Sequential + confirmatory STRATIFY testing	51.2% converted to positive; treatment modifications reported	Conference abstract
Giovannini et al. (14)	Italy	Real-world cohort	Multicentre retrospective	160	Paired (same day or ≤1 month)	Weighted κ = 0.34 (95% CI 0.23–0.45)	Conference abstract
Inojosa et al. (15)	Germany	Case-based correspondence	Letter / case report	2 cases	Cross-laboratory comparison	Illustrative examples of inter-assay discordance following biosimilar implementation	Peer-reviewed correspondence
Oreja-Guevara et al. (16)	Spain	Longitudinal cohort	Prospective longitudinal assessment	111	Repeated paired testing	45% inter-assay discrepancy; approximately 11% persistent discordance	Conference abstract
Polovina et al. (17)	Italy	Real-world cohort	Cross-sectional	111	Parallel	46.8% risk-category discordance; 48 upward versus 2 downward reclassifications	Conference abstract
Zanghì et al. (18)	Italy	Real-world cohort	Cross-sectional	69	Parallel	85.5% agreement; weighted κ = 0.62	Peer-reviewed
Fernández-Espigares et al. (19)	Spain	Real-world cohort	Cross-sectional	48	Parallel	JCV seropositivity 33.3% versus 4.2%; PABAK 0.33; minimal impact on high-risk categorisation	Peer-reviewed

JCV, John Cunningham virus; κ, Cohen’s kappa coefficient; PABAK, prevalence-adjusted bias-adjusted kappa.

Only studies providing direct comparative data between STRATIFY and IMMUNOWELL assays in natalizumab-treated patients with multiple sclerosis were included. Study categories were assigned according to the primary methodological objective of each report. Diagnostic performance measures represent inter-assay agreement using STRATIFY as comparator and do not constitute validation against clinical PML outcomes or an independent biological gold standard. Conference abstracts and preprints were included because of the recent introduction of IMMUNOWELL and the limited availability of fully published comparative datasets. When potentially overlapping datasets were subsequently reported as peer-reviewed publications, the journal publication was prioritised whenever possible.

The included evidence comprised 8 peer-reviewed publications, 7 conference abstracts and 1 preprint, encompassing analytical validation studies, parallel comparative cohorts, longitudinal studies, and post-switch observational datasets.

The available evidence encompasses several methodological approaches (**Figure 1**). Two studies provided analytical validation data assessing inter-assay performance characteristics under controlled laboratory conditions (10, 11). Real-world observational studies included both cross-sectional cohorts using paired or same-day parallel testing designs (4–6, 12, 14, 17–19) and sequential post-switch cohorts conducted after transition from originator to biosimilar natalizumab (7–9, 13).

Cross-sectional studies generally compared STRATIFY and IMMUNOWELL results obtained from the same sample or from samples collected at the same time point, thereby minimizing the influence of biological variation over time. In contrast, post-switch studies assessed IMMUNOWELL results after replacement of STRATIFY testing in routine practice and therefore incorporated variable intervals between determinations, potentially reflecting both analytical differences and temporal fluctuations in anti-JCV antibody levels.

In the UK post-switch cohort reported by Varley et al. (7), the median interval between STRATIFY and IMMUNOWELL determinations was 151 days. One Spanish cohort provided longitudinal data with repeated paired testing over time, allowing assessment of inter-assay variability across serial measurements (16). In addition, a brief case-based report illustrated examples of inter-assay discordance and highlighted methodological considerations arising after biosimilar implementation (15).

When overlapping datasets were subsequently published as full peer-reviewed articles, journal versions were prioritized over conference abstracts. An exception was made for the expanded Varley dataset presented at ECTRIMS (8), which reported a larger sample size and additional follow-up data compared with the corresponding publication (7) and was therefore retained.

Key study characteristics, testing designs, sample sizes and principal findings are summarized in **Table 1**.

### 3.2 Overall JCV seropositivity according to assay

Across comparative studies, IMMUNOWELL consistently yielded higher assay-defined JCV seropositivity rates than STRATIFY, although the magnitude of this difference varied substantially across cohorts and study designs (4–7, 9).

In analytical validation studies, differences in seropositivity were generally modest and largely attributable to assay calibration characteristics and cut-off selection (10, 11). In contrast, larger differences were observed in real-world cohorts, particularly in studies evaluating patients after implementation of biosimilar natalizumab (4–7, 9).

Several studies reported substantial increases in assay-defined seropositivity with IMMUNOWELL. In the Dutch cohort reported by Gelissen et al. (6), JCV seropositivity increased from 22.3% with STRATIFY to 55.4% with IMMUNOWELL. Similarly, Vukusic et al. (5) reported seropositivity rates of 23.6% and 61.4% respectively, while Høgestøl et al. (9) observed an increase from 13% to 52% following assay replacement in routine clinical practice. In a Spanish cohort, seropositivity increased from 4.2% with STRATIFY to 33.3% with IMMUNOWELL (19).

Across studies, the higher positivity rate observed with IMMUNOWELL was predominantly driven by patients classified as seronegative by STRATIFY who were subsequently classified as seropositive by IMMUNOWELL (4, 5, 7). The magnitude of divergence was greater in cohorts enriched for lower antibody index values, whereas concordance improved at higher index ranges and in populations with higher baseline seroprevalence (6, 9). Because available studies included both paired same-sample comparisons and sequential post-switch designs, observed differences likely reflect varying contributions of analytical classification characteristics and temporal biological variability. The wide variation in reported seropositivity rates partly reflects differences in study design and patient populations (**Table 1**).

### 3.3 Qualitative agreement and direction of discordance

Reported binary discordance between STRATIFY and IMMUNOWELL ranged from 5.8% to 37.8%, across studies, depending on cohort characteristics, antibody index distribution, and testing design (4, 5, 7, 13, 19). Discordant classifications were observed in both same-sample or same-day paired comparisons and sequential post-switch designs.

Across studies, discordance demonstrated a consistent directional pattern with IMMUNOWELL-positive/STRATIFY-negative classifications occurring substantially more frequently than the reverse scenario, which was uncommon and generally confined to predefined index cut-offs (4, 5, 19). In sequential post-switch cohorts focusing on previously classified as STRATIFY-negative, reclassification to IMMUNOWELL-positive status ranged from 47.6% to 58.7% (7, 8, 13).

Although discordance was observed across all study designs, its interpretation differs according to methodology. In paired same-sample or same-day comparisons, discordance primarily reflects analytical differences between assays. In contrast, sequential post-switch studies may capture both analytical variability and biological fluctuations in anti-JCV antibody levels occurring over time, particularly when substantial intervals separate determinations (7–9, 16).

When PML risk categories were analyzed rather than binary serostatus, discrepancies increased substantially. In a large multicenter cohort, discordance in categorical risk assignment reached 42.9% (5), illustrating how relatively small quantitative differences around index thresholds may translate into different risk strata and potentially influence clinical decision-making.

Agreement metrics ranged from substantial to moderate. Cohen’s κ reached 0.86 in a cohort with high overall concordance (12) and was approximately 0.62 in cohorts enriched for low-index values (18). Reduced agreement was consistently concentrated within the lower antibody index range, where classification thresholds exert greater influence on categorical assignment (6, 17). In the Spanish cohort reported by Fernández-Espigares et al. (19), overallbinary agreement was 66.7% (PABAK 0.33), and discordance was predominantly directional, with upward reclassification from STRATIFY-negative to low- or intermediate-risk IMMUNOWELL categories. Notably, only one patient experienced a change in high-risk status.

### 3.4 Quantitative differences in antibody index values

Quantitative comparisons performed on paired or identical samples consistently demonstrated slightly higher anti-JCV antibody index values with IMMUNOWELL than with STRATIFY (6, 7, 12, 18). Although the magnitude of these differences varied across cohorts, they were most evident within the lower index range, where positive IMMUNOWELL classifications frequently corresponded to values just above the assay-specific positivity threshold, whereas corresponding STRATIFY values often remained below the positivity cut-off or close to zero (6, 7, 18).

Several studies reported that discordant results clustered predominantly within the lower quantitative spectrum, particularly around assay-specific decision thresholds. In the UK cohort reported by Varley et al. (7), most discordant cases occurred among patients with low antibody index values, while concordance increased substantially at higher index levels. Similar observations were reported in Italian and Spanish cohorts, where discordance was concentrated among patients with lower antibody index values and became progressively less frequent with increasing antibody indices (17–19).

This distribution pattern suggests that inter-assay variability primarily affects classification close to decision thresholds rather than the overall quantitative range. Conversely, patients with clearly elevated antibody indices generally showed greater concordance across assays and more stable risk categorization (7, 12, 18). Longitudinal quantitative data remain relatively limited. However, available studies indicate that fluctuations and reversions are most commonly observed among patients with low-positive IMMUNOWELL results, whereas higher antibody index values appear to demonstrate greater temporal stability across repeated assessments (8, 16, 19).

### 3.5 Diagnostic performance metrics

When STRATIFY was used as the reference comparator, IMMUNOWELL consistently demonstrated high sensitivity and negative predictive value (NPV) across both analytical validation and real-world studies. These findings reflect strong inter-assay agreement for negative classifications rather than validated biological sensitivity. In analytical validation, sensitivity was 97.2%, specificity 74.1%, positive predictive value (PPV) 83.5%, and NPV 95.3% (10).

In real-world cohorts, sensitivity and NPV remained very high, whereas specificity and PPV were lower. In a Dutch cohort, IMMUNOWELL showed a sensitivity and NPV of 100%, with a specificity of 57.5% and PPV of 40.4% (6). In a large UK cohort (n = 497), sensitivity was 98.8% and NPV 97.8%, whereas specificity and PPV were 52.4% and 67.3%, respectively (7).

These estimates represent conditional agreement relative to STRATIFY and should not be interpreted as measures of diagnostic accuracy against an independent biological gold standard. Whether higher assay-defined seropositivity with IMMUNOWELL reflects increased analytical sensitivity, reduced specificity, or differences in threshold calibration relative to underlying biological JCV exposure cannot be determined from inter-assay comparisons alone.

Lower specificity and PPV were predominantly observed within the low-index range, where most discordant cases corresponded to upward reclassification from negative to low-positive categories (6, 7, 17, 19). Additional estimates reported in smaller cohorts showed greater variability, largely reflecting the statistical instability of performance metrics in a setting with very low STRATIFY-defined seropositivity (19). Collectively, these findings reinforce that sensitivity, specificity, PPV and NPV should be interpreted primarily as measures of inter-assay agreement rather than biological diagnostic performance.

### 3.6 Reclassification across PML risk categories

Inter-assay differences translated into clinically relevant shifts in PML risk categorization across multiple cohorts. Although the magnitude of reclassification varied according to study design and population characteristics, IMMUNOWELL consistently assigned a greater proportion of patients to higher risk categories than STRATIFY (5, 17–19).

In a large French cohort, 42.9% of patients exhibited discordance in categorical risk assignment, with IMMUNOWELL more frequently allocating patients to higher risk strata (5). Of these, 3.9% were reclassified to high risk and 10.8% would have met predefined criteria for treatment discontinuation if conventional risk thresholds had been applied.

Similarly, in an Italian cohort, 52 patients (46.8%) showed discordant risk classifications, with 48 being reclassified to higher categories by IMMUNOWELL compared with only two by STRATIFY (17).

Comparable findings were reported in additional Italian studies. In one cohort, overall agreement in risk category assignment was 85.5% (κ = 0.62); however, the proportion of patients classified as intermediate or high risk was substantially greater with IMMUNOWELL (21.7%) than with STRATIFY (8.7%) (18). In the Spanish cohort, reclassification predominantly affected low- and intermediate-risk categories, whereas changes in high-risk assignment were uncommon, with only one patient experiencing a change in high-risk status (19).

Across studies, upward reclassification was concentrated among patients with low STRATIFY antibody index values. This pattern mirrors the distribution of inter-assay discordance observed in quantitative analyses and suggests that relatively small differences around assay-specific thresholds may translate into substantially different risk classifications. Although sequential post-switch studies cannot fully exclude changes in anti-JCV antibody status over time, the consistent concentration of discordance near lower index ranges across both paired and sequential designs supports a substantial contribution of assay-related variability to categorical instability.

### 3.7 Longitudinal observations and repeat testing

Longitudinal and repeat-testing studies provided important insights into the temporal behaviour of discordant anti-JC virus antibody results. Across cohorts, fluctuations and reversions were most frequently observed among patients with lower IMMUNOWELL index values, whereas higher antibody index levels generally demonstrated greater stability over time. Fluctuations were most frequently observed in patients with index values close to assay cut-offs (7, 8, 16, 19).

In a Spanish longitudinal cohort, 45% of patients with two sequential determinations exhibited inter-assay discrepancies, with persistent discordance documented in approximately 11% of cases (16). Discordant results clustered predominantly within the lower antibody index range, particularly around assay-specific decision thresholds. Similarly, repeated measurements demonstrated that fluctuations between positive and negative classifications occurred primarily among patients with low-index IMMUNOWELL results, whereas sustained high-index classifications showed greater reproducibility across assays (16, 19).

Additional longitudinal observations from the UK post-switch cohort further highlighted the dynamic nature of low-positive results. In the expanded ECTRIMS dataset reported by Varley et al. (8), approximately 19.5% of patients who initially tested IMMUNOWELL-positive subsequently reverted to STRATIFY-negative status on follow-up testing. These reversions occurred predominantly within the low-index range and support the interpretation that a proportion of discordant results may reflect classification instability near assay-specific thresholds rather than sustained biological changes. Although longitudinal evidence remains limited, the available data consistently indicate that persistence, trajectory, and repeatability provide clinically relevant information beyond a single antibody measurement. Collectively, these findings support cautious interpretation of isolated low- positive IMMUNOWELL results and underscore the value of repeat testing when discordant classifications arise near decision thresholds.

### 3.8 Reported changes in clinical management

Several real-world studies reported changes in clinical management following reassessment of anti-JCV antibody status with IMMUNOWELL. Although the type and frequency of these interventions varied across cohorts and healthcare settings, upward reclassification was frequently associated with modifications in monitoring strategies and, in some cases, therapeutic decisions (4, 5, 7, 13, 19).

In an Italian sequential cohort, 41 of 75 patients (51.2%) were reclassified as JCV-positive with IMMUNOWELL, most within the low-index range (13). Following reassessment, 21 patients adopted extended-interval dosing and 7 transitioned to anti-CD20 therapies. Similarly, in a large French cohort, inter-assay differences resulted in clinically relevant changes in risk categorization, with 3.9% of patients being reclassified to high-risk status and 10.8% meeting predefined criteria for natalizumab discontinuation based solely on categorical reassignment (5).

In the UK post-switch cohort, assay reclassification was associated with increased MRI surveillance, additional clinical consultations, and treatment modifications in a subset of patients (7). Other studies reported that upward reclassification influenced monitoring intensity and longer-term therapeutic planning, particularly among patients with lower antibody index values (4, 19).

Although available evidence is predominantly observational and several reports derive from conference abstracts, the consistency of these findings across independent cohorts suggests that inter-assay variability may have tangible consequences for patient management. These observations highlight the importance of cautious interpretation of low-positive results and support the integration of longitudinal data and broader clinical context into decision-making processes, particularly when discordant results occur near assay-specific thresholds.

## 4 Discussion

The present review highlights a challenge that extends beyond simple analytical discordance between anti-JCV antibody assays. The principal issue emerging from the available evidence is the application of PML risk models that were originally derived and validated within a single-assay framework to a clinical environment in which multiple assays now coexist. Natalizumab-associated PML risk stratification algorithms were developed using STRATIFY-derived antibody index values and calibrated within a mono-assay ecosystem (1, 2). The introduction of IMMUNOWELL in the context of biosimilar natalizumab implementation has created a multi-assay environment in which quantitative values, positivity thresholds, and categorical risk assignments may no longer be directly interchangeable. The central observation emerging from the available literature is that the transition from a mono-assay to a multi-assay environment represents a previously underrecognized source of variability in PML risk stratification. This variability arises not only from differences in assay performance, but also from the transfer of risk models developed within one analytical framework to a setting in which multiple platforms coexist. To our knowledge, this review provides one of the first structured syntheses examining inter-assay variability not only as a laboratory concordance issue, but as a structural challenge affecting the transferability of established PML risk models across analytical platforms.

Across analytical validation studies and real-world cohorts, inter-assay differences become most apparent near predefined decision boundaries, where relatively small quantitative variations may translate into substantial categorical reclassification (4–9, 17–19). Importantly, discordance is not uniformly distributed across the antibody index continuum. Rather, it clusters predominantly within lower quantitative ranges and around assay-specific cut-offs (6–8, 17–19). When identical or paired samples are analysed, IMMUNOWELL generally yields modestly higher numerical index values than STRATIFY, particularly near the positivity threshold (6–8, 10, 18). These observations support the concept of a cut-off proximity instability zone, in which minor analytical variation is disproportionately amplified by binary classification systems. Such instability does not necessarily imply biological change but reflects the interaction between continuous quantitative measurements and rigid categorical thresholds. The phenomenon becomes particularly evident once more than one analytical platform is implemented in routine practice.

From an immunological and analytical perspective, both STRATIFY and IMMUNOWELL measure anti-JCV IgG responses, yet they differ in antigen composition, assay calibration procedures, confirmatory testing strategies, and positivity thresholds (4–11). These differences may influence the detection and classification of low-level humoral responses, particularly near assay-specific decision boundaries. Consequently, weak antibody reactivity that is classified as negative by one platform may be reported as low-positive by another, contributing to the observed concentration of discordance within lower index ranges. This does not necessarily imply analytical inadequacy of either assay; rather, it illustrates how different analytical systems may classify weak or borderline antibody signals differently. Whether discordant low-level reactivity reflects biologically meaningful anti-JCV humoral responses, analytical detection of weak antibody signals, or a combination of both remains uncertain.

A further consideration relates to study design. The available literature includes both same-sample or same-day parallel comparisons and sequential post-switch cohorts performed after replacement of STRATIFY by IMMUNOWELL in routine clinical practice. Paired studies provide the most direct assessment of analytical differences between assays because both measurements are obtained from the same sample or from samples collected at the same time point. In contrast, sequential designs may also capture biological fluctuations in anti-JCV antibody levels occurring over time. Therefore, part of the discordance reported in post-switch studies may reflect temporal biological variability rather than purely analytical factors. Nevertheless, the consistent concentration of discordance within low-index ranges across both methodological approaches strengthens the observation that assay-related factors contribute substantially to the reported variability (7–9, 16).

These findings should not be interpreted as evidence of analytical inadequacy of IMMUNOWELL. Across analytical validation studies and real-world studies, IMMUNOWELL demonstrated high sensitivity and preserved negative predictive value when STRATIFY was used as the reference comparator (6, 7, 10). However, because no independent biological gold standard exists for latent JCV infection, sensitivity and specificity estimates derived from inter-assay comparisons should be interpreted as measures of agreement rather than true diagnostic accuracy. Whether higher IMMUNOWELL seropositivity reflects enhanced analytical sensitivity, reduced specificity, differences in threshold calibration, or a combination of these factors remains uncertain. This distinction is essential, because STRATIFY functions as the historical comparator for established PML risk algorithms, but it does not represent an absolute biological reference standard for latent JCV infection.

Longitudinal observations provide additional insight into the potential biological significance of discordant results. Across available cohorts, fluctuations and reversions were observed predominantly among low-positive IMMUNOWELL classifications, whereas higher antibody index values demonstrated greater temporal stability and inter-assay concordance (7, 8, 16, 19). The recently published Spanish cohort further illustrates this pattern, showing substantial differences in overall seropositivity but limited impact on high-risk categorization (19). Collectively, these findings suggest that persistence and trajectory may provide clinically relevant information beyond that obtained from a single isolated measurement near an assay-specific threshold. In practical terms, a newly detected low-positive result close to the cut-off should not necessarily be interpreted in the same way as a persistent or rising antibody index over serial determinations.

**Figure 2:**
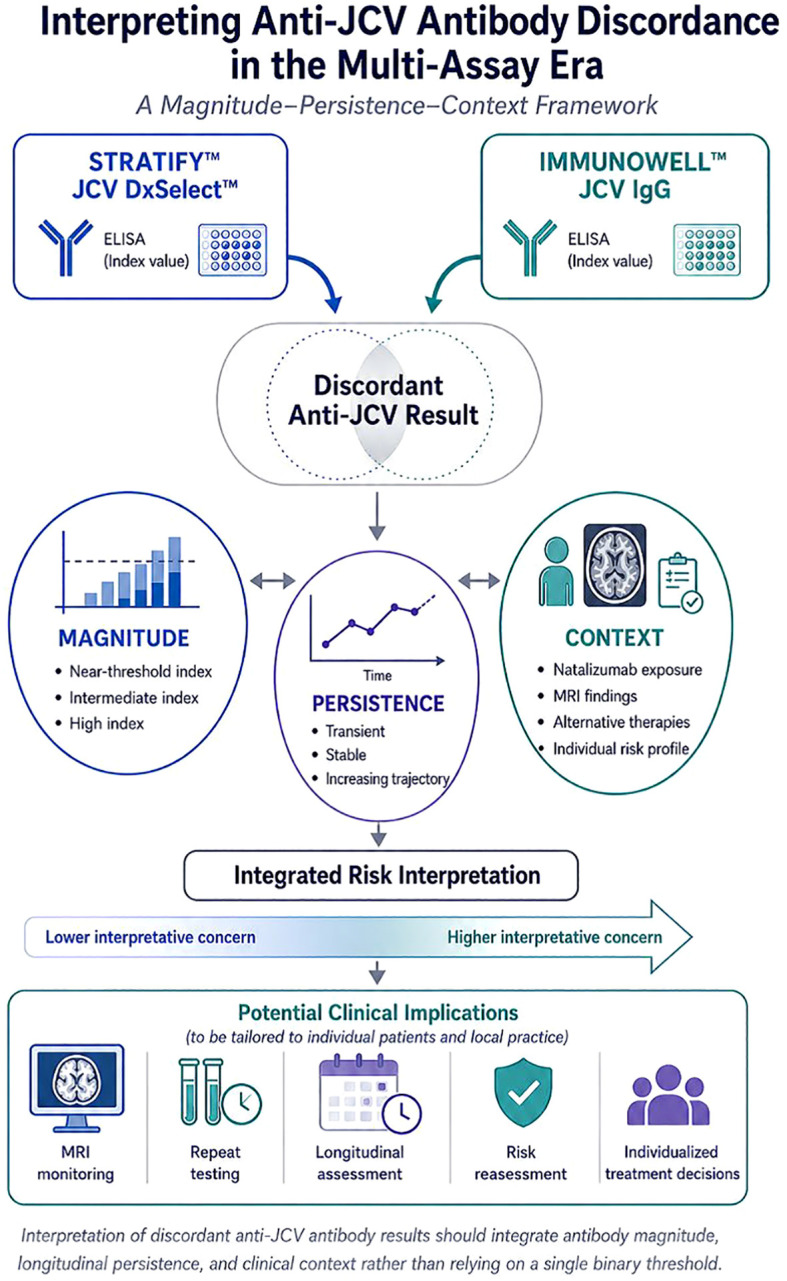
Magnitude–persistence–context framework for interpretation of discordant anti-JCV antibody results in a multi-assay environment. Conceptual framework illustrating a contextual approach to the interpretation of discordant anti-JCV antibody results obtained with different commercial assays. Rather than relying exclusively on binary classifications, interpretation incorporates three complementary dimensions: antibody magnitude, longitudinal persistence, and overall clinical context. These elements contribute to an integrated assessment of interpretative concern and may inform potential clinical implications in individual patients. The framework is exploratory, derived from patterns consistently observed across comparative studies, and is not intended as a validated clinical algorithm or a substitute for established PML risk stratification models.

Taken together, the available evidence indicates that discordant anti-JCV antibody should not be interpreted solely through a binary lens. Instead, interpretation may benefit from a framework integrating antibody magnitude, longitudinal persistence, and overall clinical context. **Figure 2** summarizes this proposed magnitude–persistence–context framework and illustrates how discordant results may be interpreted within a continuum of clinical concern rather than through a single positive/negative threshold. Importantly, **Figure 2** should be viewed as a conceptual framework intended to support clinical interpretation rather than as a validated clinical algorithm. This approach aims to distinguish between low-level, potentially unstable results near assay cut-offs and more reproducible high-index profiles historically associated with increased PML risk.

On the basis of patterns consistently observed across comparative studies, we also propose an exploratory descriptive interpretative framework together with practical scenarios summarized in **Table 2**. The scenarios in **Table 2** illustrate how these principles may be applied across commonly encountered patterns of inter-assay discordance, including concordant negative results, low-index upward reclassification, persistent low-positive discordance, concordant high-index results, and upward shifts toward intermediate or high-risk categories. Importantly, this framework should be regarded as an exploratory conceptual model rather than a validated clinical algorithm. Its purpose is not to replace established PML risk stratification tools, but to support structured clinical reasoning when discordant results arise within a multi-assay environment.

**Table 2:** Descriptive framework for contextual interpretation of discordant anti–JCV antibody results in a multi-assay environment.

Scenario	STRATIFY result	IMMUNOWELL result	Typical index range	Interpretative consideration	Suggested clinical approach	Supporting references
Concordant negative	Negative	Negative	Below assay-specific cut-off	Stable low antibody signal	Routine monitoring	(6, 7, 17)
Low-index upward reclassification	Negative	Low positive	Around assay-specific cut-offs	Cut-off proximity instability; possible analytical variability	Repeat testing before escalation	(6–8, 17, 19)
Persistent low-positive discordance	Negative	Low positive (repeated)	Around assay-specific cut-offs	Uncertain biological significance; requires longitudinal evaluation	Monitor trajectory and clinical context	(16, 17, 19)
Concordant high index	High	High	Clearly above high-risk thresholds	Stable high antibody signal; historically associated with increased PML risk	Apply established high-risk algorithms	(1, 2, 7)
Upward shift to intermediate/high	Low / intermediate	Intermediate / high	Near risk-category thresholds	Quantitative divergence near threshold	Confirm longitudinal persistence before major change	(5, 18, 19)

This framework is exploratory and intended to support contextual interpretation of discordant anti-JCV antibody results in a multi-assay environment. It represents a conceptual synthesis of patterns consistently observed across comparative studies and should not be interpreted as a validated clinical algorithm or a substitute for established PML risk stratification models. The proposed scenarios are intended to facilitate clinical reasoning by integrating antibody magnitude, longitudinal persistence, and overall clinical context, consistent with the conceptual framework illustrated in **Figure 2**. Prospective outcome-based validation is currently unavailable.

IMMUNOWELL, IMMUNOWELL™ JCV; JCV, John Cunningham virus; PML, progressive multifocal leukoencephalopathy; STRATIFY, STRATIFY JCV™ DxSelect™.

Although the biological implications of low-level discordance remain uncertain, the practical consequences are increasingly evident. Several cohorts reported intensified MRI surveillance, adoption of extended-interval dosing, additional clinical assessments, and treatment modifications following assay-related reclassification (5, 7, 13). These observations indicate that inter-assay variability may influence clinician behaviour, patient perception, and healthcare resource utilization even in the absence of demonstrated increases in observed PML incidence. Recent expert guidance addressing JCV testing in the biosimilar era similarly recommends cautious interpretation of newly detected low-positive results, particularly when discordant with previous STRATIFY findings (20). In this respect, the present review complements rather than duplicates consensus-based guidance: while expert consensus provides pragmatic clinical recommendations, our synthesis seeks to explain why discordance occurs, where it is most likely to arise, and how it may be conceptualized within the broader transition from a mono-assay to a multi-assay risk-stratification environment.

Future research should focus on assay harmonization initiatives, calibration studies aimed at defining assay-specific risk thresholds, and prospective multicentre registries incorporating longitudinal anti-JCV antibody trajectories. Particular emphasis should be placed on outcome-based validation studies linking assay-derived classifications with clinically adjudicated PML events. Such efforts will be essential to determine whether observed inter-assay differences translate into meaningful differences in clinical risk and to support the development of robust risk-stratification frameworks within an increasingly multi-assay environment.

This review has several limitations. First, the available evidence remains heterogeneous and includes analytical validation studies, observational cohorts, conference abstracts, and one preprint. Importantly, a substantial proportion of the currently available literature derives from congress abstracts that have not undergone full peer review and frequently provide limited methodological detail, incomplete follow-up, or preliminary analyses. Furthermore, findings reported in conference abstracts may evolve following full peer-reviewed publication and should therefore be interpreted cautiously. Although the overall findings were generally consistent across studies, conclusions derived from peer-reviewed publications should be considered more robust than those based exclusively on conference reports. To minimize this limitation, potentially overlapping datasets were carefully assessed and peer-reviewed publications were prioritised whenever available. Second, methodological heterogeneity, including the coexistence of paired and sequential testing approaches, may contribute to variability in reported discordance rates. Third, prospective outcome studies linking IMMUNOWELL-derived antibody index trajectories with adjudicated PML events are not yet available, limiting definitive conclusions regarding the clinical significance of low-level discordance. Future validation efforts will need to integrate both longitudinal antibody trajectories and clinically adjudicated PML outcomes to determine the clinical significance of assay-related discordance. Finally, because contemporary PML incidence has declined substantially in the setting of established risk mitigation strategies, very large longitudinal datasets will be required to calibrate assay-specific risk models against clinical outcomes. Consequently, the findings of this review should be interpreted as hypothesis-generating rather than outcome-validating.

## 5 Conclusions

IMMUNOWELL and STRATIFY do not provide fully interchangeable classifications in patients with MS treated with natalizumab. Across comparative studies, IMMUNOWELL consistently assigns a higher proportion of assay-defined JCV seropositivity, with discordance occurring predominantly among low index values close to assay-specific decision thresholds. Agreement improves at higher index levels, and changes in high-risk assignment are less frequent than changes within low- or intermediate-risk categories.

The findings reviewed here suggest that the principal challenge introduced by the coexistence of multiple anti-JCV antibody assays is not merely analytical discordance itself, but the application of risk models developed within a single-assay framework to a multi-assay clinical environment. In this context, interpretation of discordant results may benefit from consideration of antibody magnitude, longitudinal persistence, and overall clinical context rather than reliance on isolated binary classifications.

Pending prospective outcome-based validation and assay-specific risk calibration, cautious interpretation of newly detected low-positive or discordant results remains warranted. Repeat testing, longitudinal assessment, and integration of clinical factors may help contextualize assay-related variability within established PML risk mitigation strategies. As JCV serology evolves from a mono-assay to a multi-assay environment, future risk stratification strategies will likely require greater emphasis on quantitative trajectories and contextual interpretation rather than reliance on assay-specific binary thresholds alone.
